# Prevalence of the Relative Age Effect in Elite Brazilian Volleyball: An Analysis Based on Gender, the Playing Position, and Performance Indicators

**DOI:** 10.2478/hukin-2022-0093

**Published:** 2022-11-08

**Authors:** Henrique de Oliveira Castro, Samuel da Silva Aguiar, Lucas Savassi Figueiredo, Lorenzo Laporta, Gustavo De Conti Teixeira Costa, José Afonso, Sérgio Adriano Gomes, Vivian de Oliveira

**Affiliations:** 1 Physical Education Department, Federal University of Mato Grosso, Cuiabá, Brazil; 2 Physical Education Department, University Center UDF, Brasília, Distrito Federal, Brazil; 3 Physical Education Department, Federal University of Juiz de Fora – Governador Valadares Advanced Campus, Governador Valadares, Brazil; 4 Center for Advanced Study and Research in Sports, Center of Physical Education and Sport, Federal University of Santa Maria, Santa Maria, Brazil; 5 Center for Advanced Study and Research in Sports, Faculty of Physical Education and Dance, Federal University of Goiás, Goiânia, Brazil; 6 Centre for Research, Education, Innovation, and Intervention in Sport, University of Porto, Porto, Portugal; 7 Graduate Program in Physical Education, Catholic University of Brasília, Taguatinga, Brazil; 8 Physical Education Department, University Center UniCEUB, Brasília, Brazil; 9 Physical Education Department, University Center IESB, Brasília, Brazil; 10 Bioscience Institute, São Paulo State University, Rio Claro, Brazil

**Keywords:** talent selection, selection bias, athletes

## Abstract

Athletes born closer to an arbitrary cut-off date are more likely to reach an elite level in sport, which is supported by a phenomenon called the relative age effect (RAE). It is important to determine whether this phenomenon is present in a sport to minimize this selection bias. This study aimed to investigate the prevalence of RAE in elite volleyball athletes, considering the influence of gender, the playing position (Setter, Middle, Libero, Opposite, and Outside Hitter) and the performance level (attack points, aces, and block points). The sample comprised 203 male and 193 female athletes competing in the Superliga A in the 2020/2021 season, which was equivalent to all of the teams of the championship. The data collection was performed during May and June, 2021. Athletes were organized according to gender, the playing position, and performance in the Superliga. For performance variables, athletes were separated based on the median value (90.0), and classified as high- or low-performance. Chi-squared tests were performed to verify differences between birth date distributions in relation to the aforementioned variables. Results indicated overrepresentation of relatively older male athletes (Q1 = 35.96%; Q2 = 27.59%; Q3 = 19.21%; Q4 = 17.24%), especially in Middles, Opposites, and Outside Hitters, regardless of their performance level. Considering females, no differences were found. Our findings suggest that RAE operates differently for men and women in elite Brazilian volleyball. The characteristics of the games played by male and female elite athletes may lead to different talent selection processes, affecting the likelihood of RAE prevalence.

## Introduction

The phenomenon of relative age effect (RAE) reflects the asymmetrical distribution of athletes based on their birth dates, relative to an arbitrary cutoff (Lidor et al., 2021). Evidence indicates that RAE influences selection and talent identification in different team sports (Rubajczyk and Rokita, 2020; Schorer et al., 2013) such as soccer (Yagüe et al., 2020; Figueiredo et al., 2022; Szwarc et al., 2019), basketball (Kalén et al., 2021), rugby (Kelly et al., 2021), futsal (Castro et al., 2022), handball (de la Rubia et al., 2021; Peña-González et al., 2021), and volleyball (Campos et al., 2020; Işın. and Melekoğlu 2020; Rubajczyk and Rokita, 2020; Safranyos et al., 2019; Solon Junior and Silva Neto, 2020), with older athletes having better chances of being selected by coaches and talent scouts compared to their younger peers within an age category (Furley and Memmert, 2016).

One of the explanations of this phenomenon is based on differences between athletes’ maturational stages. This is because relatively older athletes may be more likely to enter puberty earlier and exhibit advanced physical characteristics (Cobley et al., 2009; Musch and Grondin, 2001). In this sense, athletes born in the beginning of the year could have transient performance advantages such as increased height, muscle mass, aerobic power, muscle strength, endurance and speed. These qualities are important for many sports (Malina et al., 2015), and may affect the coaches perception of talent (Cobley et al., 2009; Furley and Memmert, 2016), favoring a biased selection process that upholds relatively older athletes.

Nevertheless, in order to understand RAE it is necessary to consider other factors. Aiming to explain the RAE phenomenon, Wattie et al. (2015) proposed a constraint-based model that accounted for the interaction between individual (e.g., sex, height, body composition, maturational status), environment (e.g., sport's popularity, its policies, and physical environment, among others), and task (sports specificity, such as the competitive level and physical capabilities that are more important for success) constraints in a given sport’s system. Specifically at the elite level, RAE seems to play a major role in the recruitment of individuals in sports, affecting the opportunities they may receive to develop their athletic skills (Lidor et al., 2021).

Even though RAE is more pronounced in youth sports (Musch and Grondim, 2001), evidence indicates that RAE may remain up to adult-elite professional players in some contexts (Joyner et al., 2020; Yagüe et al., 2020). On the other hand, evidence indicates that in other contexts RAE decreases (Lupo et al., 2019) or even reverses (Joyner et al., 2020; de la Rubia et al., 2020) in the transition to elite sport. These conflicting results reinforce the multifactorial nature of RAE and indicate the need to investigate this phenomenon considering the specificities of the modality in a given context, taking into account that the existence of this phenomenon can influence the opportunities given to youth athletes to reach the elite categories.

Volleyball is a team net ball sport with an intermittent type of effort and characterized by unpredictability that involves a combination of repetitive jumps, multidirectional movements, and long, extended matches, in which both teams play on separate courts, with the possession of the ball changing cyclically during the rally (Lima et al., 2020). Brazil has a long tradition in volleyball, which may affect the presence of RAE (Musch and Grondin, 2001), since the social context, the level of competition, the popularity of a sport, and the number of active participants may increase the competition for spots in clubs.

Thus, considering the specific characteristics of the sport system in a given country is warranted in new studies focused on RAE (Lupo et al., 2019). Many studies have investigated RAE in volleyball, considering different sport levels, such as youth athletes (Campos et al., 2020; Okazaki et al., 2011; Rubajczyk and Rokita, 2020), university athletes (Safranyos et al., 2019), and senior athletes who participated in World Championships (Solon Junior and Silva Neto, 2020), with controversial results. With regard to elite Brazilian volleyball athletes, only one study, to our knowledge, has assessed RAE among male and female athletes who competed in the National League first division (Parma and Penna, 2018). However, this study’s analysis was restricted to the birth quartiles of these athletes, not accounting for other factors that may have influenced RAE in this context.

Considering the relevance of RAE to talent identification, this study investigated the prevalence of RAE in Brazilian elite volleyball athletes who participated in the Superliga A in the 2020/2021 season, considering their playing position (Setters, Middles, Liberos, Opposites, and Outside Hitters), gender (male or female), and performance in the League (attack points, aces, and block points). We hypothesized that RAE would be found regardless of gender, the playing position and performance, due to the elite context being analyzed.

## Methods

### Participants

The sample was composed of 396 elite volleyball athletes (male: n = 203, mean age = 24.6 ± 6 years; female: n = 193, mean age = 26.1 ± 6.1 years), which was equivalent to all athletes of the 12 male and 12 female teams that competed in the Superliga A (first division) in the 2020/2021 season. The adopted exclusion criteria included incomplete data, which resulted in the exclusion of four male and five female athletes from the study. The athletes were organized according to their gender, playing position, and performance in the Superliga.

To analyze the performance in Superliga A 2020/2021, statistics provided by the Brazilian Volleyball Confederation (CBV) were used, consisting of the attack points (attack points), direct service points (aces), and direct blocking points (block points). These actions were chosen because they represent offensive actions which most often culminate in direct points. For organization and analysis of performance variables, athletes were separated based on the median value (median = 90.0). Athletes who surpassed the median were considered high-performance players, while those who did not surpass the median value were considered low-performance ones (Castro et al., 2022).

### Data collection and procedures

This study adopted similar methods to those applied previously in volleyball (Parma and Penna, 2018). Data were obtained from the CBV official website (www.cbv.com.br) the most important institution of volleyball in Brazil and the organizer of the competition, which guarantees the reliability of data. Data collection was performed during May and June 2021. The information obtained included players’ full names, gender, date of birth, playing position, and performance in the Superliga A 2020/2021, considering attack points scored, aces, and block points.

We defined the birth year as beginning on January 1^st^, as used by other studies in team sports (Castro et al., 2022). Data were tabulated in a spreadsheet, and variables analyzed included the quarters of the year athletes were born (Castro et al., 2022; Parma and Penna, 2018) as follows: Q1 = first quarter (January to March); Q2 = second quarter (April to June); Q3 = third quarter (July to September); and Q4 = fourth quarter (October to December). Additionally, players were categorized according to gender (male and female), playing position (Setter, Middle, Libero, Opposite, and Outside Hitter), and performance (attack points, aces, and block points) in the Superliga A 2020-2021.

### Statistical analysis

The chi-square test (χ2) was performed to verify differences between birth date distributions in relation to gender, playing position and performance, as previously proposed in recent research that investigated RAE in volleyball (Campos et al., 2020; Rubajczyk and Rokita, 2020; Solon Junior and Silva Neto, 2020). Athletes were divided into four quarters (Q1, Q2, Q3, and Q4), and 25% was assumed as the expected frequency for each quarter, as proposed by Cotê et al. (2006). When the observed distributions were different from the expected, post hoc comparisons were performed between quarters, to determine in which quarters athletes’ frequencies were different from the expected. In these cases, we adjusted the significance level to 0.0083 through the Bonferroni’s correction. In addition, the same analysis was carried out considering the birthdate distribution in each semester (1^st^ sem.: January to June and 2^nd^ sem.: July-December), in which 50% was assumed as the expected distribution for each semester. Whenever chi-squared test results were significant, corrected residuals (*z*-value) were calculated according to Beasley and Schumacher (1995). When the z-value was greater than |1.96| the difference for that specific cell was considered significant. Finally, the odds ratio (OR) for Q1 versus Q4 and 1^st^ versus 2^nd^ was calculated. Analyses were performed using the Statistical Package for Social Sciences (SPSS), version 21.0 (IBM^®^, Chicago, USA). The level of significance was set at *p* < 0.05.

## Results

Table 1 shows the absolute distribution of birthdates of the studied elite male volleyball players. Results indicated overrepresentation of players born in the first quarters of the year (Q1 = 35.96%; Q2 = 27.59%), as well as a reduced frequency of players born closer to the end of the year (Q3 = 19.21%; Q4 = 17.24%), with significant differences (*p* < 0.05) between Q1 and Q2 compared to Q3 and Q4. Likewise, the chi-square test revealed that the distribution of the first semester was significantly higher (*p* < 0.05) compared to the second semester. On the other hand, for elite female volleyball players, no difference was found (*p* > 0.05) between birth trimesters and semesters (Table 2).

**Table 1:** Quarter of birth distribution of male Brazilian elite volleyball athletes.

Quarter of date of birth	Athletes (n)		Percentage (%)
Q1	73		35.96
Q2	56		27.59
Q3	39^a^		19.21
Q4	35^a,b^		17.24
1^st^	129		63.55
2^nd^	74^c^		36.45
χ^2^		17.906	
*df*		3	
*p*-value		<0.0001	
OR (Q1:Q4)		2.085	
OR (1^st^:2^nd^)		1.743	

*Q1*–*Q4 = birth quarter; χ2 = Chi-square value; OR = odds ratio; Q1:Q4 = first quarter compared to the fourth quarter; 1^st^:2^nd^ odds ratio from the 1^st^ semester to the 2^nd^ semester; a = pairwise Chi-square comparison different from Q1 (adjusted for multiple comparison); b = pairwise Chi-square comparison different from Q2 (adjusted for multiple comparison); c = different from the 1^st^ semester*.

**Table 2:** Quarter of birth distribution of female Brazilian elite volleyball athletes.

Quarter of the date of birth	Athletes (n)		Percentage (%)
Q1	54		27.55
Q2	55		28.06
Q3	43		21.94
Q4	44		22.45
1^st^	109		55.61
2^nd^	87		44.39
χ^2^		2.490	
*df*		3	
*p*-value		<0.477	
OR (Q1:Q4)		1.227	
OR (1^st^:2^nd^)		1.252	

*Q1*–*Q4 = birth quarter; χ2 = Chi-square value; OR = odds ratio; Q1:Q4 = first quarter compared to the fourth quarter; 1^st^:2^nd^ odds ratio from the 1^st^ semester to the 2^nd^ semester*

Table 2 shows the birthdate distribution depending on the playing position for male athletes. Uneven distributions were found for Outside Hitters, Opposites, and Middles, with more players represented (*p* < 0.05) in Q1 compared to Q3 and Q4 for Outside Hitters, Q1 and Q2 compared to Q4 for Opposites, and Q1 and Q2 compared to Q3 for Middles. There were no differences (*p* > 0.05) in the distribution of birthdates for Liberos and Setters. Considering female players, no differences were found (*p* > 0.05) in the birthdate’s distribution according to the playing position (Table 4).

**Table 3:** Distribution of birthdates in male athletes according to the playing position.

	Q1			Q2			Q3			Q4							
						
Variable	n	%	*z*-value	n	%	*z*-value	n	%	*z*-value	n	%	*z*-value	χ2	*df*	*p*-value	OR (Q1:Q4)	OR (1^st^:2^nd^)
**Playing Position**													8.265	12	0.764		
Outside Hitters	24	32.9	0.5	16	28.6	-0.4	12^a^	30.8	0.0	10^a^	28.6	-0.3	7.419	3	0.060	2.40	1.82
Opposites	11	15.1	0.4	9	16.1	0.6	6	15.4	0.3	2^a,b^	5.7	-1.5	6.571	3	0.087	5.50	2.50
Liberos	6	8.2	-1.0	7	12.5	0.3	6	15.4	0.9	4	11.4	0.0	0.826	3	0.843	1.50	1.30
Setters	10	13.7	-1.0	8	14.3	-0.7	9	22.9	1.1	8	22.9	1.0	0.314	3	0.957	1.25	1.06
Middles	22	30.1	0.7	16	28.6	0.3	6^a,b^	15.4	-1.8	11	31.4	0.6	10.236	3	0.017*	2.00	2.24

*Q1*–Q4 = birth quarter; χ2 = Chi-square value; OR = odds ratio; Q1:Q4 = first quarter compared to the fourth quarter; 1^st^:2^nd^ odds ratio from the 1^st^ semester to the 2^nd^ semester; a = pairwise Chi-square comparison different from Q1 (adjusted for multiple comparison); b = pairwise Chi-square comparison different from Q2 (adjusted for multiple comparison); * = significant difference.

**Table 4:** Distribution of birthdates in female athletes according to the playing position.

	Q1			Q2			Q3			Q4							
						
Variable	n	%	*z*-value	n	%	*z*-value	n	%	*z*-value	n	%	*z*-value	χ2	*df*	*p*-value	OR(Q1:Q4)	OR(1^st^:2^nd^)
**Playing Position**													6.466	12	0.891		
Outside Hitters	13	24.1	-1.1	20	36.4	1.2	12	27.9	-0.4	14	31.8	0.3	2.627	3	0.453	0.93	0.40
Opposites	7	13.0	0.1	6	10.9	-0.5	5	11.6	-0.3	7	15.9	0.7	0.440	3	0.932	1.00	1.08
Liberos	7	13.0	0.1	8	14.5	0.5	5	11.6	-0.3	5	11.4	-0.3	1.080	3	0.782	1.4	1.50
Setters	13	24.1	1.5	5	9.1	-1.9	8	18.6	0.2	8	18.2	0.2	3.882	3	0.274	1.6	1.12
Middles	14	25.9	-0.2	16	29.1	0.4	10	30.2	0.5	10	22.7	-0.7	1.415	3	0.702	1.3	1.30

*Q1*–*Q4 = birth quarter; χ2 = Chi-square value; OR = odds ratio; Q1:Q4 = first quarter compared to the fourth quarter; 1^st^:2^nd^ odds ratio from the 1^st^ semester to the 2^nd^ semester*.

Table 5 shows the male athletes’ birthdate distribution according to their performance (low and high) in attack points, aces, and block points. Uneven distributions were found for players with low performance, with overrepresentation of (*p* < 0.05) Q1 and Q2 compared to Q4 in all variables (attack points, aces, and block points), and Q1 and Q2 compared to Q3 for aces. Regarding players with high performance, unequal distributions were also found, with overrepresentation (*p* < 0.05) of Q1 compared to Q3 and Q4 in all variables (attack points, aces, and block points). When female athletes were analyzed, chi-square tests did not find statistically significant differences (*p* > 0.05) in the birthdate distribution regardless of the performance level (Table 6).

**Table 5:** Distribution of birthdates in male athletes according to performance.

	Q1			Q2			Q3			Q4							
						
Variable	n	%	*z*- value	n	%	*z*- value	n	%	*z*- value	n	%	valu *z*- e	χ2	*df*	*p*- value	OR (Q1:Q4)	OR (1^st^:2^nd^)
**Attack Points**													1.171	3	0.760		
Low	32	47.8	-0.6	30	56.6	1.0	19	50.0	-0.1	14^a,b^	46.7	-0.5	9.463	3	0.024*	2.28	1.87
High	35	52.2	0.6	23	43.4	-1.0	19^a^	50.0	0.1	16^a^	53.3	0.5	8.978	3	0.030*	2.18	1.65
**Aces**													2.853	3	0.307		
Low	33	49.3	-0.3	30	56.6	1.0	16^a,b^	42.1	-1.2	16^a,b^	53.3	0.3	10.305	3	0.016*	2.06	1.96
High	34	50.7	0.3	23	43.4	-1.0	22	57.9	1.2	14^a^	46.7	-0.3	8.720	3	0.033*	2.42	1.58
**Block Points**													1.376	3	0.623		
Low	31	46.3	-0.9	29	54.7	0.7	19	50.0	-0.1	16^a,b^	53.3	0.3	6.853	3	0.077	1.93	1.71
High	36	53.7	0.9	24	45.3	-0.7	19^a^	50.0	0.1	14^a^	46.7	-0.3	11.473	3	0.009*	2.57	1.82

*Q1*–Q4 = birth quarter; χ2 = Chi-square value; OR = odds ratio; Q1:Q4 = first quarter compared to the fourth quarter; 1^st^:2^nd^ odds ratio from the 1^st^ semester to the 2^nd^ semester; a = pairwise Chi-square comparison different from Q1 (adjusted for multiple comparison); b = pairwise Chi-square comparison different from Q2 (adjusted for multiple comparison); * = significant difference.

**Table 6:** Distribution of birthdates in female athletes according to performance.

	Q1			Q2			Q3			Q4							
						
Variable	n	%	*z*- value	n	%	*z*- value	n	%	*z*- value	n	%	*z*- value	χ2	*df*	*p*- value	OR (Q1:Q4)	OR (1^st^:2^nd^)
**Attack Points**													2.925	3	0.403		
Low	20	40.8	-1.5	27	54.0	0.7	19	47.5	-0.3	24	57.1	1.1	1.822	3	0.610	0.83	1.09
High	29	59.2	1.5	23	46.0	-0.7	21	52.5	0.3	18	42.9	-1.1	2.846	3	0.416	1.61	1.33
**Aces**													2.316	3	0.509		
Low	24	49.0	-0.1	27	54.0	0.7	16	40.0	-1.4	23	54.8	0.7	2.889	3	0.409	1.04	1.31
High	25	51.0	0.1	23	46.0	-0.7	24	60.0	1.4	19	45.2	-0.7	0.912	3	0.823	1.31	1.12
**Block Points**													0.195	3	0.978		
Low	24	49.0	-0.1	26	52.0	0.4	19	47.5	-0.3	21	50.0	0.0	1.289	3	0.732	1.14	1.25
High	25	51.0	0.1	24	48.0	-0.4	21	52.5	0.3	21	50.0	0.0	0.560	3	0.905	1.19	1.17

*Q1*–*Q4 = birth quarter; χ2 = Chi-square value; OR = odds ratio; Q1:Q4 = first quarter compared to the fourth quarter; 1^st^:2^nd^ odds ratio from the 1^st^ semester to the 2^nd^ semester*

## Discussion

The present study aimed to investigate the existence of RAE in Brazilian elite volleyball athletes who participated in the Superliga A 2020/2021, according to their gender, playing position, and performance in the League. Overall, our results indicated that the prevalence of RAE in elite Brazilian volleyball was modulated by gender and the playing position in male athletes, which partially confirms our hypothesis. However, no RAE was found in females, which contradicts our initial expectations. These findings are discussed below, considering the literature on RAE.

Gender analysis revealed that RAE was prevalent in male, but not in female elite Brazilian volleyball athletes. The results found in our male sample corroborate the notion that RAE can be pervasive in some contexts and it remains at the same level in the adult category (Cobley et al., 2009). Similar results were reported by Lupo et al. (2019), who analyzed the initial phase of the career of 3,319 adult athletes from various team sports, and found that relatively older athletes were overrepresented in the early stage of the sports career, corroborating that RAE may prevail up to adults when it is very pervasive in the youth context. On the other hand, Solon Junior and Silva Neto (2020) did not find any RAE in Olympic volleyball players, speculating that complex factors such as refined tactical skills may reduce the impact of maturational aspects. Our results contradict the findings of Solon Junior and Silva Neto (2020), since RAE was present in the analyzed elite male athletes. These differences could be explained by characteristics of this sport context, in which the maturational aspects are determinant for the selection of talents in youth categories (Campos et al., 2020), favoring relatively older athletes who usually present an anticipated maturation process (height and muscle mass) compared to younger athletes (Malina et al., 2015). This affects the athletes’ opportunities in training and competition settings, increasing the likelihood of relative older male athletes reaching the adult category of Brazilian elite volleyball (Parma and Penna, 2018).

No RAE was found in the analyzed female volleyball athletes, which is in line with previous research that verified the absence of RAE in female elite adult athletes from various sports (Smith et al., 2018). Similar results were reported by Parma and Penna (2018) in an analysis of athletes participating in the Brazilian Women's Volleyball Superliga (2016/2017). Since the distribution of birthdates was balanced among this pool of athletes, it suggests that RAE is not pervasive in the women’s Brazilian elite volleyball. It could be assumed that RAE is absent in this specific sports context because female athletes tend to mature earlier than male athletes (Malina et al., 2015). This makes it more likely that coaches rely on other aspects besides physical factors (e.g., technical and tactical performance) to determine the selection and permanence of female athletes in youth sport, since maturational aspects are expected to be more homogenous when these selection processes occur, reducing the chances of RAE (Helsen et al., 2007). Research that investigates the presence of RAE in international (Campos et al., 2020), and Brazilian (Okazaki et al., 2011) female youth elite athletes seems to support this notion. It is important to highlight that this is a speculative argument, since we did not analyze data from Brazilian volleyball youth categories.

Analyses of playing positions indicated that relatively older athletes were overrepresented in male Outside Hitters, Opposites, and Middles, but not in Liberos and Setters. These findings are in line with evidence from other modalities indicating that playing positions modulate the occurrence of RAE in elite team sports (Figueiredo et al., 2022). Considering specifically male volleyball, playing positions in which RAE was present are more physically and anthropometrically demanding than positions in which no RAE was found. Indeed, Outside Hitters, Opposites, and Middles perform more blocking and spiking actions, which are highly dependent on physical capacities such as body height, spike reach and, block reach (Palao et al., 2014). On the other hand, Liberos and Setters perform more receiving, digging and setting actions, which are less dependent on physical features. With regard to female athletes, the lack of differences among all playing positions was somehow expected, and our results indicated that RAE was not prevalent in the female sample of this study.

In our study RAE was also analyzed according to performance indicators. However, performance did not seem to modulate RAE in Brazilian elite volleyball, since RAE was found in male, but not female athletes regardless of the performance level. Our findings regarding male athletes differ from those reported by Solon Junior and Silva Neto (2020), in which performance indicators were not influenced by RAE in Olympic male athletes. However, these findings are in line with our data on female athletes. On the other hand, Campos et al. (2020) found a higher proportion of female World Championship medalists born in the first months of the year, indicating an indirect relationship between RAE and performance, corroborating the results found in male athletes. Considering females, no RAE was found regardless of the performance level. As previously stated, these results indicated that RAE was not prevalent in elite Brazilian women’s volleyball. The divergence between our results and previous findings reinforces the need to continue investigating the relationship between RAE and performance indicators in volleyball.

In summary, our findings indicate that RAE was pervasive in male elite Brazilian volleyball, but not in female. It is possible that characteristics of the games played by men and women influenced these results. While the male elite volleyball game is more influenced by anthropometric and physical aspects, the female elite volleyball game is characterized by continuous actions, relying more on technical and tactical aspects (Costa et al., 2012). For this reason, it is possible that Brazilian youth volleyball coaches conduct their talent selection processes considering these specificities. This would result in a selection process that is more focused on anthropometric and physical aspects in male youth athletes, favoring the occurrence of RAE. On the other hand, it is more likely that the selection of female youth athletes relies more on technical and tactical aspects, reducing the likelihood of RAE. Further research that investigates the criteria used by Brazilian volleyball coaches and the prevalence of RAE in youth categories of Brazilian volleyball is necessary to confirm such hypotheses.

This study has several limitations, such as the reliability of data as we relied on the statistics provided by the CBV. Another limitation of the study were the performance measures considered. Since we based our performance analysis only on attack points, aces, and block points, it is possible that setters and libero players were at a disadvantage, because attackers are expected to score most of the points, and this may have accentuated RAE for these players. Future investigations should propose specific performance indicators for libero and setter positions, reducing the eventual bias created by the measures used in our study.

Our findings have practical implications and reinforce the need to make coaches and sport administrators aware of the existence of RAE in Brazilian volleyball, specifically in male athletes who play at Middle, Opposite, and Outside Hitter positions. We speculate that the selection of these athletes in the youth categories is affected by the maturational confound, which is troublesome, since RAE and maturation coexist and operate differently in the talent selection process (Hill et al., 2020). In this sense, strategies to reduce the disadvantages faced by relative younger athletes in the selection processes during youth categories are warranted to minimize the systematic loss of potential sporting talents in the long run (Figueiredo et al., 2022). Applying a minimum of quotas for each birth quarter in youth competitions and the use of age-ordered shirt numbering in youth selection processes to make chronological differences more evident (Mann and van Ginneken, 2017) are some of the proposed alternatives meant to reduce RAE in sports.

In summary, our findings indicate that RAE is prevalent in male Brazilian elite volleyball players, but not in female players. Regarding male players, this effect is modulated by playing positions, since relatively older players are more frequent in Outside Hitters, Opposites, and Middles. Therefore, coaches and sport administrators are warranted to apply counter-RAE interventions in male youth volleyball, to reduce the inequalities generated by RAE, specifically in the aforementioned positions.
